# Antipruritic Effect of Acupuncture in Patients with Atopic Dermatitis: Feasibility Study Protocol for a Randomised, Sham-Controlled Trial

**DOI:** 10.1155/2017/1926806

**Published:** 2017-11-14

**Authors:** Yu-Kang Kim, Mijung Yeom, SeHyun Kang, Hi-Joon Park, Kyuseok Kim, Hyangsook Lee

**Affiliations:** ^1^Department of Korean Medical Science, Graduate School, Kyung Hee University, 26 Kyungheedae-ro, Dongdaemun-gu, Seoul 130-701, Republic of Korea; ^2^Acupuncture & Meridian Science Research Centre, College of Korean Medicine, Kyung Hee University, 26 Kyungheedae-ro, Dongdaemun-gu, Seoul 130-701, Republic of Korea; ^3^Department of Ophthalmology, Otorhinolaryngology and Dermatology of Korean Medicine, College of Korean Medicine, Kyung Hee University, 26 Kyungheedae-ro, Dongdaemun-gu, Seoul 130-701, Republic of Korea; ^4^Australian Research Centre in Complementary and Integrative Medicine, Faculty of Health, University of Technology Sydney, Sydney, NSW 2007, Australia

## Abstract

This study aims to test the feasibility of a randomised clinical trial to evaluate how acupuncture affects atopic dermatitis (AD) symptoms and quality of life and to explore potential biomarkers that may be associated with AD. It is a sham-controlled trial in which 30 eligible patients will be randomly allocated in a 1 : 1 : 1 ratio to one of three groups: verum acupuncture (VA) group 1 (3 times weekly for 4 weeks); VA group 2 (twice weekly for 4 weeks); or sham acupuncture group (SA; twice weekly for 4 weeks). SA will consist of nonpenetrating acupuncture. Outcome measures will include the Visual Analogue Scale for itch, SCORing Atopic Dermatitis, and Eczema Area and Severity Index to evaluate AD symptoms improvement along with the Patient Oriented Eczema Measure and Dermatology Life Quality Index to assess quality of life. Measures will be collected at baseline, once weekly during the treatment period, and after a 4-week follow-up period. Blood collection will be at baseline and 4 and 8 weeks after treatment and compared with healthy controls. Illumina sequencing will be used to profile microRNA expression in each group to explore candidate microRNA biomarkers for specific effects of acupuncture in patients with AD. This trial is registered via US National Institutes of Health Clinical Trials registry (ClinicalTrials.gov) on 15 July 2016, identifier: NCT02844452.

## 1. Background

The prevalence of atopic dermatitis (AD), a chronic relapsing inflammatory skin disease characterized by eczematous and intensely pruritic skin lesions [1, 2], is increasing worldwide [3, 4]. In South Korea, about 13% of children and 3% of adults were reported to be diagnosed with AD in a 2008–2011 survey [5, 6]. Pruritus associated with AD often induces sleeping problems and patients with AD frequently complain of a lowered health-related quality of life; for example, daily activities and/or personal relationships may be restricted [7].

First-line therapy of AD is based on the use of emollients and other systemic and/or topical treatments: topical corticosteroid (TCS) and calcineurin inhibitors are recommended as topical management; antimicrobials, systemic immune-modulators, allergen-specific immunotherapy, and phototherapy are used for systemic management [8–11]. However, other complementary and alternative therapies are commonly used by many patients with AD when conventional treatments yield unsatisfactory relief or produce side effects [12].

In Korea, patients with AD have frequently used Traditional Korean Medicine, such as acupuncture and herbal medicine, for symptom management [13]; however, there have been only a small number of clinical studies of AD treatment with acupuncture and herbal medicine and even fewer studies investigating the therapeutic effect of acupuncture alone [12, 14]. This may explain why a recent systematic review failed to include any randomised clinical trials (RCTs) of acupuncture as treatment of AD [15]. Nevertheless, previous reports have demonstrated that acupuncture exerts an antipruritic effect on pruritogen-induced scratching behaviour in an animal model and on allergen-induced itch in patients with AD [16–19]. Considering its potential antipruritic effect, this highlights the need for rigorously designed and conducted trials in acupuncture field [15].

To overcome some weaknesses of previous studies and to determine whether acupuncture helps AD symptom management, we will conduct a randomised, sham-controlled feasibility trial comparing two different doses of active acupuncture treatment with a sham acupuncture control. We aim to establish the feasibility of recruitment and to determine key features, including optimal acupuncture treatment dose and outcome measures, to inform a large-scale RCT in patients with AD. Additionally, we will explore novel biomarker candidates that may be associated with the antipruritic effect of acupuncture.

## 2. Materials and Methods

### 2.1. Objectives

The aims of this study are (1) to investigate the feasibility of a large-scale randomised clinical trial to test the antipruritic effect of acupuncture treatment compared with a sham acupuncture in patients with AD; (2) to identify the optimal dose and schedule of acupuncture treatment and to identify outcome measures that will reflect symptoms changes; and (3) to discover biomarkers that may be associated with the therapeutic effects of acupuncture.

### 2.2. Hypotheses

1. Acupuncture treatment will mitigate pruritus and other symptoms of AD and will improve quality of life in patients with AD, compared with sham acupuncture.
2. Three times weekly acupuncture treatment over one month will produce better outcomes than twice weekly acupuncture treatment over one month.
3. Certain biomarker levels might be affected by the therapeutic effect of acupuncture and/or development of AD.

### 2.3. Trial Design

**Figure 1:**
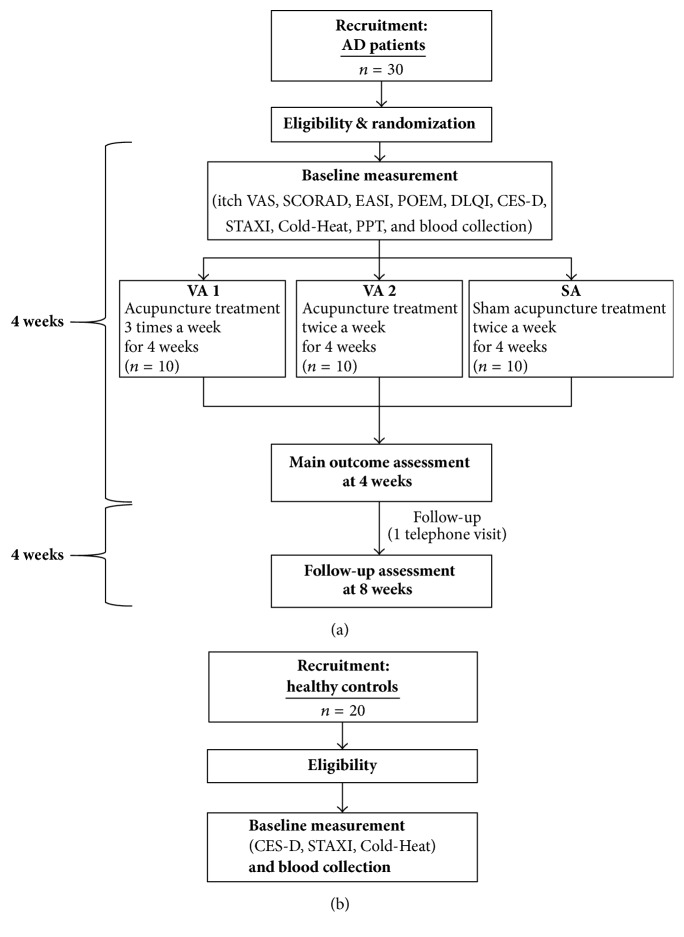
*Flow of the trial*. (a) Randomisation schema for participants with AD. (b) Recruitment of healthy controls for comparative microRNA expression profiling analysis against AD patients. AD, atopic dermatitis; CES-D, Centre for Epidemiologic Studies-Depression Scale; Cold-Heat, questionnaire of cold-heat pattern identification based on diagnosis in Traditional Korean Medicine; DLQI, Dermatology Life Quality Index; EASI, Eczema Area and Severity Index; POEM, Patient Oriented Eczema Measure; PPT, Pressure Pain Threshold; SCORAD, SCORing Atopic Dermatitis; STAXI, state-trait anger expression inventory; VAS, Visual Analogue Scale.

A randomised, sham-controlled feasibility trial will be conducted at the Kyung Hee University Korean Medicine Hospital from June 2016 to April 2017 (Figure 1(a)). In addition to patients with AD, healthy volunteers will be recruited to compare various biomarkers between the groups (Figure 1(b)).

### 2.4. Participants and Recruitment

#### 2.4.1. Patients with AD

*(1) Inclusion Criteria*. Participants who meet all of the following criteria will be included:

1. Age 19 and older
2. Meeting the Hanifin and Rajka criteria [20], with AD symptoms appearing constantly for at least 3 months prior to study enrolment
3. Score > 3 points and <8 points on itch Visual Analogue Scale (VAS): 1–10 scale
4. SCORing Atopic Dermatitis (SCORAD) index score 10–40
5. Not taking prescription drugs for AD for at least 1 month prior to enrolment
6. Ability to understand the study protocol and to voluntarily agree to participate
7. Not participating in any other research studies for AD for at least 1 month prior to enrolment.

*(2) Exclusion Criteria*. Participants who meet any of the following criteria will be excluded:

1. Severely fluctuating AD symptoms
2. Treatments that are not allowed in this study, for example, oral corticosteroids, which may affect the study outcomes
3. Asthma or bronchitis
4. Other disorders that may affect the outcomes, for example, anxiety or depression.

#### 2.4.2. Healthy Controls

*(1) Inclusion Criteria*. Participants who meet all of the following criteria will be included:

1. Age 19 and older
2. No present or past history of allergic diseases such as AD, asthma, or allergic rhinitis
3. Ability to understand the study protocol and to voluntarily agree to participate.

*(2) Exclusion Criteria*. Participants who meet any of the following will be excluded:

1. Pregnant or lactating women
2. History of anaphylaxis, severe hypertension, hypotension, or respiratory disease
3. Regular use of antihistamines or steroids during the previous month.

### 2.5. Recruitment

Advertisements will be posted on notice boards of the hospital and local community centres.

### 2.6. Randomisation and Allocation Concealment

After signing the written consent, participants will undergo a screening test to confirm eligibility. The baseline characteristics including demographics, physical examination, medical history, and severity of AD signs will be used to screen the eligible participants according to the inclusion and exclusion criteria. Individuals with AD will be randomly assigned to one of three groups via block randomisation. Healthy volunteers will not be assigned to a treatment group (Figure 1).

An independent statistician will produce random numbers with the PROC PLAN of SAS 9.2 (SAS Institute Inc., Cary, NC, USA) and transfer them to the Acupuncture and Meridian Science Research Centre (AMSRC) at Kyung Hee University, where each random number will be sealed in a sequentially numbered opaque envelope by a staff member who is otherwise not involved in this study.

At the beginning of the randomisation process, the investigator will call the independent staff in the AMSRC to receive the screening number of the participant. Then the AMSRC staff screening ID and treatment assignment will be documented in the trial master file.

### 2.7. Blinding and Code Breaking

Participants and the outcome assessor will be blinded to the treatment allocation in this trial. Since it is impossible to blind the Korean Medicine Doctors (KMDs) who will give acupuncture treatments, they will be excluded from performing the outcome measurements or analysing data. To ensure participant blinding, an eye patch will be applied to patients during the acupuncture treatment, and they will be unable to see the practice procedures. All the acupuncture treatments, regardless of group assignment, will utilise the Park sham device (AcuPrime Co., Ltd., Exeter, UK), which is applied at the acupoints to block participants from seeing whether or not the acupuncture needles penetrate the skin [21]. The acupuncture needles and device will be maintained in place for 15 min in all treatment groups. If a participant has a serious adverse event and immediate cessation of acupuncture treatment is required, the blinding code will be broken.

### 2.8. Interventions

#### 2.8.1. Acupuncture Treatment

The acupuncture treatments were based on the revised STandards for Reporting Interventions in Clinical Trials of Acupuncture (STRICTA) 2010 recommendations (Table 1) [22]. Both manual acupuncture and intradermal acupuncture treatment will be given. The acupuncture protocols reflect everyday clinical practice in Korean Medicine.

In this study, manual acupuncture treatment is composed of fixed acupuncture treatment and additional acupuncture treatment: partially individualised acupuncture treatment will be performed on the basis of the traditional meridian theory and consensus by experts in acupuncture and AD. The number of applied acupoints per patient per session ranges from 6 to 19: 6 basic points and 10 optional points (3 optional points can be applied bilaterally). All participants with AD will receive acupuncture needling at 6 fixed points: LI11, ST36, and PC6, bilaterally. The optional points include ST43, GB41, LI2, TE3, SI3, TE6, SI2, BL66, LR3, and SP3. The optional acupoints will be added in accordance with individual signs or symptoms, contralaterally to the applied signs or symptoms: ST43 and GB41 for gastric stuffiness or dyspepsia; LI2 and GB41 for tenderness around ST25, diarrhoea, or constipation; TE3 and SI3 for fullness in the chest and hypochondrium; TE3 and TE6 for lower abdominal pain plus tenderness on the chest centre; SI3 and GB41 or SI2 and BL66 for lower abdominal pain, dry skin, or heat in the upper body and cold in the lower body; and LR3 and SP3 for pain in hypogastric region with darkness of the sublingual collateral vessels. A study coordinator will ask participants about symptoms that might require additional acupuncture treatment to reduce the bias. Regardless of group assignment, all manual acupuncture treatment will be performed using Park sham devices to maintain participant blinding. The acupuncture treatment will be done with disposable sterilised stainless needles (40 mm length and 0.25 mm diameter; Dongbang Acupuncture Inc., Bundang, Seongnam, Korea) or Park sham acupuncture needles (AcuPrime Co., Ltd., Exeter, UK) according to the assigned groups. Manipulation technique will be performed to elicit “de qi” sensation. The needle will be inserted up to 5 to 30 mm depending on acupoints and retained for 15 minutes.

After removal of needles, intradermal needles using 1.5 mm press tack needles (Haeng Lim Seo Won Medical Co., Korea) will be given to three acupoints per participant per session: LI11 bilaterally and auricular-Shenmen contralaterally. This is aimed at mitigating AD-related pruritus with LI11 and reducing anxiety level with auricular-Shenmen [16, 23, 24]. Participants will be instructed by study staff to press the applied press tack needles located on LI11 for 3 minutes when they feel severe pruritus.

All acupuncture treatments will be carried out by the same KMD, who was trained for 10 hours to guarantee that he could perform the acupuncture treatments identically according to the predefined protocol.

#### 2.8.2. Verum Acupuncture Group 1 (VA1)

Participants assigned to the VA1 will receive 12 acupuncture sessions over 4 weeks, that is, 3 days/week. A telephone interview will take place two weeks after the end of treatment to collect data on the severity of pruritus symptoms, amount of TCS used, and adverse effects. Participants' final visit will occur 4 weeks after the end of treatment. No other intervention will be permitted during the study period.

#### 2.8.3. Verum Acupuncture Group 2 (VA2)

Participants assigned to the VA2 will receive 8 acupuncture sessions over 4 weeks, that is, 2 days/week. A telephone interview will be given two weeks after the end of treatment to collect data on the severity of pruritus symptoms, amount of TCS used, and adverse effects. Participants' final visit will occur 4 weeks after the end of treatment. No other intervention will be permitted during the study period.

#### 2.8.4. Sham Acupuncture Group (SA)

Participants assigned to the SA group will receive 8 acupuncture sessions over 4 weeks, that is, 2 days/week. Sham acupuncture treatment will be conducted on 6 control points: a point 1 to 2 cm proximal and 1 cm medial to LI7, a point 1 cm proximal and 1 cm medial to LI11, and a point 1 cm proximal and 1 cm lateral to ST36, each bilaterally. Instead of intradermal needles, the same sized stainless steel rings without needles, that is, nonpenetrating intradermal needles, which were designed and validated for blinding for our study, will be attached to three control points: a point 1 cm proximal and 1 cm medial to LI11, bilaterally, and finger point in the ear, contralaterally [25]. Unlike VA1 and VA2, fixed acupuncture treatments will be given, but questions about symptoms, TCS, and adverse effects will be asked to participants in this group. A telephone interview will also be given two weeks after the end of treatment to collect data on the severity of pruritus symptoms, amount of TCS used, and adverse effects. After the 4-week treatment period, participants will return for a final visit at 4 weeks after the end of treatment, identical to VA1 and VA2.

### 2.9. Compliance and Discontinuation

During the study period, each patient will receive either 12 or 8 treatment sessions according to their group assignment. Since frequent hospital visits may be the burden to participants and may induce them to withdraw from the trial, we identified a minimum number of treatments to be considered compliant, as follows: at least 10 sessions of 12 should be completed for VA1, and at least 6 sessions of 8 should be completed for VA2 and SA. For dropouts who did not fulfil the aforementioned compliance criteria, last available data will be taken for the analysis.

The study coordinator will maintain regular contact and encourage participants to complete all treatment sessions and follow-up visits. Should a participant withdraw consent, any data collected from the participant will be kept in files safely for 3 years and then will be discarded.

### 2.10. Outcome Measures

One research nurse who is otherwise not involved in this study will conduct a series of outcome measures: its schedule is depicted in Table 2. There will be no separation between primary and secondary outcomes since it is an exploratory study to evaluate the independent effectiveness of acupuncture to treat AD and to collect outcome data for further study.

Participants with AD will complete all the outcome measures; healthy controls will submit to blood collection and complete the Cold-Heat, Centre for Epidemiologic Studies-Depression Scale (CES-D), and state-trait anger expression inventory (STAXI) questionnaires.

#### 2.10.1. SCORAD Index

SCORAD is a widely used tool for assessing severity of AD. The SCORAD evaluates the intensity and extent of affected regions and estimates subjective discomfort such as pruritus and sleep loss symptoms [26–28]. The SCORAD index contains 6 items to evaluate AD intensity: erythema, excoriation, oedema or papulation, lichenification, oozing or crust, and dryness. The extent of the lesions is evaluated as a percentage of entire external surface of the human body with the rule of nine. The SCORAD grades the intensity and extent of affected skin and the participant rates their subjective symptoms with VAS (0 = no itch or sleep loss, to 10 = worst imaginable itch or sleep loss).

#### 2.10.2. Itch VAS

The itch VAS is one of the components of the SCORAD index, and it will be particularly applied to assess the severity and reduction of pruritus in this trial. Participants whose itch VAS score is less than 30 or higher than 80 will be excluded because we assumed that they will have lower desire for antipruritic therapy or need more potent treatment [29].

#### 2.10.3. EASI Score

The Eczema Area and Severity Index (EASI) is a validated instrument measuring AD severity [28, 30]. Questions are specific to four body regions (head and neck, trunk, upper extremities, and lower extremities) and four symptoms (erythema, oedema/papulation, excoriation, and lichenification).

#### 2.10.4. POEM

The Patient Oriented Eczema Measure (POEM) is a useful, 7-item tool for monitoring AD severity, which focuses on participant-reported symptoms and illness. Scores range from 0 to 28, and it is suitable for administration in the outpatient clinic or in the clinical trial setting [28, 31].

#### 2.10.5. DLQI

The Dermatology Life Quality Index (DLQI) is a compact questionnaire which is applicable to individuals with any skin disease. It measures the influence of AD on participant's life over the previous 7 days. It consists of 10 questions with Likert-type responses and its score ranges from 0 to 30; higher scores imply greater influence on health-related quality of life [10, 32].

#### 2.10.6. TCS Use

Only Lidomex (prednisolone valeroacetate) will be permitted as a rescue medicine for severe pruritus, by consensus of the study KMD and the pharmacist at Kyung Hee University Korean Medicine Hospital. Participants will be instructed to apply TCS according to the fingertip method: the ointment will be applied on the surface area of one palm with amount from the tip of index finger to the distal skin crease [10].

Participants using TCS will report the amount used, frequency, and affected regions weekly during this trial. Ointments will also be weighed weekly before being dispensed and then again at the end of this study in order to evaluate the amount of TCS used [33].

#### 2.10.7. AD Pattern Questionnaire

To reflect a unique point of view on pattern diagnosis in Traditional Korean Medicine in an objective manner, we adopted the AD pattern questionnaire [34]. Participants with AD will be categorised as either excess or deficient syndrome by answering 20 “yes or no” type questions about their AD symptoms and pulse/tongue diagnoses. A KMD will make diagnoses based on the categorisation.

#### 2.10.8. Cold-Heat Questionnaire

Identifying cold and heat pattern is a relatively obvious and definite factor of diagnosis in Traditional Korean Medicine and the usual cold-heat pattern of patients plays an important role in choosing treatment modalities and establishing treatment plans [35]. We will use the questionnaire of cold-heat pattern identification based on usual conditions and signs. By assessing their usual cold-heat pattern, participants will be classified into one of four categories: Cold; Heat; No Cold-Heat; and Cold-Heat complex [36].

#### 2.10.9. PPT

Abdominal examination has been one of the characteristic methods for diagnosis, choosing a therapy, and predicting prognosis in Traditional Korean Medicine [37]. We will utilise an algometer to standardise and quantitatively describe the abdominal examination [38]. Using an algometer (Wagner Instruments, Greenwich, CT), one operator will apply pressure to 3 acupoints (CV17, CV12, and ST25) vertically against the surface. Pressure will be applied at a rate of 1 kg/cm^2^/sec and participants will be instructed to report the beginning of pain by saying “stop” immediately after the perception of pain is transferred from pressure. The examiner will immediately stop pressing and record the pressure pain threshold (PPT) value. The maximum PPT will be limited to 8 kg/cm^2^ in order to prevent distorted measures. At each assessment, PPT will be measured three times with the intervals of 1 minute [38, 39].

#### 2.10.10. CES-D

Since it has been reported that depression is strongly associated with AD, evaluating depressive symptoms of the patient is highly recommended [40, 41]. The CES-D is a reliable tool for nonpsychiatric populations and the validated Korean version will be used for this trial [42, 43].

#### 2.10.11. STAXI

It is widely reported that AD patients have a higher risk of depressive symptoms and a reduced capacity to cope with stress or anger [41, 44]. With a suggested relationship between depression and anger suppression in AD patients [41], assessing the anger trait will be valuable to interpret outcomes in the context of participants' responses.

STAXI is a validated measure for assessing the experience, expression, and control of anger. It has 3 subscales such as trait anger, anger-inside (anger suppression), and anger-outside (anger expression) and consists of a total of 44 questions [45]. The validated Korean STAXI will be applied for this trial [46].

#### 2.10.12. Credibility Test

To assess the credibility of the acupuncture treatment used in this study, participants will complete the credibility questionnaire at baseline, at 4 weeks (after the termination of treatment period), and at 8 weeks (after the end of follow-up period) [47, 48].

#### 2.10.13. Measuring Serum Levels of Various Markers

Various biomarkers have been reported to have different levels in patients with AD, but none has been validated as a definite biomarker [49–51]. In this study we will investigate a number of biomarkers (including IgE) for associations with immunological, inflammatory, psychological, and endocrine factors in AD.

About 4 ml of whole blood will be collected from participants at 3 time points: at baseline; 4 weeks after the completion of acupuncture treatment; and 8 weeks after the follow-up period. Healthy controls will submit to only 1 blood collection (at baseline). Blood will be centrifuged to obtain serum.

Changes in various biomarker levels from baseline to 4 weeks in each of the treatment groups will be examined. Between-group differences will be analysed to explore potential associations with the effect of acupuncture. Analysis of changes from 4 weeks to 8 weeks in group will be conducted to evaluate the sustained effect of acupuncture.

*(1) Serum Total IgE Measurement*. The role of IgE in the pathogenesis of AD is acknowledged as contributing to allergic sensitisation and indirectly supporting AD severity via sensitisation to various allergens [49]. It has been reported that anti-IgE therapy has a curative impact on AD and that it may be an affordable treatment modality [49, 50]. In this study, total IgE levels will be measured using Enzyme Linked Immunosorbent Assay (ELISA).

*(2) Serum Cytokine/Chemokines Measurement*. Based on knowledge of inflammatory mediators and autoimmune responses, a diversity of cytokines have been explored in AD and T cells, and related cytokines are also considered as potential influencing factors in the pathogenesis of AD [52–54]. For instance, overexpression of IL-4 and IL-13 in AD skin was reported and IL-4 was suggested to play a crucial role in AD development [52, 55, 56].

In this context, we will use a commercial kit (Bio-Plex Pro Human Chemokine 40-plex Panel) for the following 40 chemokines from extracted serum samples: 6Ckine/CCL21, BCA-1/CXCL13, CTACK/CCL27, ENA-78/CXCL5, Eotaxin/CCL11, Eotaxin-2/CCL24, Eotaxin-3/CCL26, Fractalkine/CX3CL1, GCP-2/CXCL6, GM-CSF, Gro-*α*/CXCL1, Gro-*β*/CXCL2, I-309/CCL1, IFN-*γ*, IL-1*β*, IL-2, IL-4, IL-6, IL-8/CXCL8, IL-10, IL-16, IP-10/CXCL10, I-TAC/CXCL11, MCP-1/CCL2, MCP-2/CCL8, MCP-3/CCL7, MCP-4/CCL13, MDC/CCL22, MIF, MIG/CXCL9, MIP-1*α*/CCL3, MIP-1*δ*/CCL15, MIP-3*α*/CCL20, MIP-3*β*/CCL19, MPIF-1/CCL23, SCYB16/CXCL16, SDF-1*α*+*β*/CXCL12, TARC/CCL17, TECK/CCL25, and TNF-*α*.

*(3) Serum Cortisol Measurement*. Cortisol, also called glucocorticoid, is a well-known stress-related hormone associated with the hypothalamic-pituitary-adrenal (HPA) axis and its mechanisms have been elucidated by analysing physiological change in stress response systems on a variety of psychological issues [57–60]. In this study, serum cortisol level will be used to assess HPA function in accordance with the stress response of participants. Total cortisol levels will be measured using ELISA.

#### 2.10.14. Exploring Biomarkers with MicroRNA Expression Profiling

AD is known as a complex genetic skin disease affected by environmental distress and resulting from abnormal immune responses as well as malfunctions of the skin barrier [50, 61]. But no specific biomarker for the diagnosis of atopic disorder has yet been identified [50, 51]. In this context, microRNA (miRNA) will be analysed in order to explore new candidates associated with the development of AD and its treatment with acupuncture.

A miRNA is a small noncoding RNA molecule. Since its function is related to eukaryotic cells, dysregulation of several specific miRNAs has been reported to be associated with several pathological processes, such as chronic lymphocytic leukemia and cardiomyopathy [62, 63]. In the study of AD, some miRNAs were proposed to be related to AD symptoms [64, 65], but none has been confirmed as a predictive or prognostic marker.

**Figure 2:**
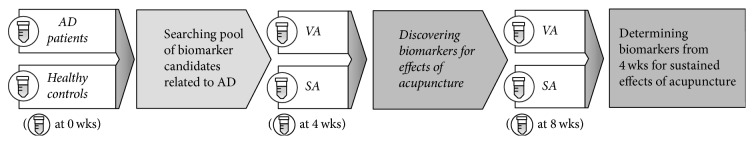
*Flow of Illumina sequencing*. AD, atopic dermatitis; VA, among VA1 and VA2, the most effective schema will be selected; SA, sham acupuncture group; wks, weeks. A tube icon means the sera obtained from the blood samples.

Illumina sequencing using serum will be performed for miRNA expression profiling to determine candidate miRNA biomarkers for the effectiveness of acupuncture. The difference between participants with AD and healthy controls at baseline will be analysed to explore potential biomarkers associated with the development of AD. Within the pool of biomarker candidates related to AD, the difference between VA1 or VA2 (whichever is more efficacious) and SA at 4 weeks will be compared, to determine biomarkers related to acupuncture effect. The difference between VA and SA at 8 weeks will also be analysed to examine whether the selected biomarkers at 4 weeks continue to be significant at 8 weeks, to explore sustained effects of acupuncture (Figure 2).

### 2.11. Statistical Methods

#### 2.11.1. Sample Size Calculation

The proposed study is a RCT whose primary aim is to investigate the feasibility to recruit and treat participants with AD with acupuncture and to evaluate acupuncture's antipruritic effects. We will calculate the effect sizes of key outcomes including itch VAS and SCORAD index, to inform power calculations for a further large-scale study. A total of 30 individuals with AD and 20 healthy controls will be recruited during the study period; participants with AD will be randomly assigned in a 1 : 1 : 1 ratio to one of two active acupuncture regimens or sham acupuncture. Healthy controls will not receive any treatment. Considering an estimated 20% dropout rate, we identified the sample size with the advice of a statistician. The statistical significance level will be set at 0.1 considering the applied small size.

#### 2.11.2. Statistical Analysis Plan

We will perform descriptive analyses for all outcome measures. Categorical variables will be provided with percentages, and continuous variables will be provided as means with standard deviations. An independent statistician will perform statistical analyses using SPSS 21.0 (IBM SPSS Statistics, New York, USA). Missing data will be replaced by carrying forward the most recent nonmissing value, and the time and reason for the missing data will be recorded. Depending on the recorded reasons, multiple imputation by an independent statistician will be done and compared.

The effectiveness of the intervention will be provided by examining changes in outcome measures between baseline and end of treatment and through the follow-up period, using a repeated measures analysis of variance (ANOVA) and *χ*^2^ test. The difference in effect between VA1 and VA2 will be analysed by examining changes in the itch VAS and SCORAD index, by the Mann–Whitney *U* test on continuous variables and *χ*^2^ test on categorical variables. The difference in therapeutic effect between VA (using the most efficacious dose between VA1 and VA2) and SA will be investigated in the same way. The data will be analysed with a *p* = 0.1 (two-sided) statistical significance level and 90% confidence intervals will be provided following the statistician's advice for a small sample size. The current data will be used to calculate the number of participants needed to reach a significance level of 0.05 and 80% power.

### 2.12. Protection of Participants

#### 2.12.1. Ethical Approval and Registration

This study protocol was approved by the ethics committee at Kyung Hee University Korean Medicine Hospital (KOMCIRB-160212-HRBR-004). It will be performed in accordance with the standards of the International Committee on Harmonisation on Good Clinical Practice and the revised version of the Declaration of Helsinki. This trial was registered via US National Institutes of Health Clinical Trials registry (ClinicalTrials.gov) on 15 July 2016 (identifier: NCT02844452). The trial registration was conducted after the start of participant recruitment due to delays in the preparation of registration.

#### 2.12.2. Participant Safety

During each visit, unanticipated problems or adverse events related to acupuncture treatment will be reported by participants and documented by study staff. Acupuncture treatment has been reported as a safe treatment rarely causing adverse effects; common adverse effects caused by acupuncture treatment include bleeding, hematoma, and pain [66]. Any unfavourable or unexpected signs related to study treatment or procedures will be compiled on paper, managed under the care of practitioners, and reported to institutional and other oversight agencies as appropriate.

#### 2.12.3. Quality Control

All participating KMDs and outcome assessors will be trained before the beginning of the trial to ensure consistent quality of practices. The training program will cover diagnoses, inclusion and exclusion criteria, location of the acupoints, acupuncture manipulation techniques, measurement of PPT, and filling out case report forms. Participants who discontinue or who withdraw consent will also be thoroughly documented. This study will be monitored by the AMSRC and periodic monitoring will ensure the accuracy and the quality of this study. We checked Standard Protocol Items: Recommendations for Interventional Trials (SPIRIT) to improve the quality of clinical trial protocols (see Additional File 1 in Supplementary Material available online at https://doi.org/10.1155/2017/1926806) [67].

#### 2.12.4. Confidentiality

After enrolment, participants' personal information will be accessible only to the investigator. Research data and blood samples will be identified by a unique code to protect confidentiality during and after the trial. All study personnel completed training in protection of personal health information.

### 2.13. Protocol Amendments

If a protocol modification is required (such as due to an unanticipated problem or adverse event), the representatives of Kyung Hee University Korean Medicine Hospital and the AMSRC will discuss the matter fully to take the appropriate steps.

## 3. Discussion

A recent systematic review failed to identify any RCTs on acupuncture as treatment for AD, as it has not been extensively researched [15]. We describe a protocol for a randomised, sham-controlled feasibility trial comparing two VA groups with different treatment schedules with SA to assess the effectiveness of acupuncture in individuals with AD, to be conducted in clinical settings in Korea. The aims are to evaluate whether the proposed acupuncture treatments warrant further investigation for improvement of symptoms, mental health, and quality of life and whether a relationship can be established between certain biomolecules and the development of AD by comparing levels in the blood of individuals with AD to healthy controls.

Determining an optimal dose of acupuncture treatment involves complex decision making [68, 69]. Clinical practice and patients' acceptability should be carefully considered when comparing two different dosages/schedules (3 days weekly versus 2 days weekly for 4 weeks). Our study results will elucidate which regimen produces maximal effectiveness and which schedule participants prefer, which is of importance to practitioners and educators.

We will limit enrolment to individuals with mild to moderate AD. Since KMDs usually treat severe cases with more intensive combinations of treatment, such as acupuncture and herbal medicine or acupuncture and hospitalisation, we selected acupuncture alone in order to detect differences between treatments without introducing other confounders; in addition, rescue medication will be permitted as we believe that this strategy reflects real world practice.

A battery of outcome measures will be used in our study since it is exploratory in nature. We will investigate the effects of acupuncture treatment on AD symptom improvement with a particular focus on antipruritic effect, measured using itch VAS, SCORAD, EASI, and POEM. Information on rescue medication use and quality of life will also be collected. For pattern identification in Korean Medicine, the AD pattern and Cold-Heat questionnaires, along with PPT, will be administered. CES-D and STAXI will examine potential anxiolytic and stress-relieving effects of acupuncture. Through credibility testing, the feasibility of participant blinding to treatment assignment will be thoroughly examined. Some may argue that nonspecific effects may unnecessarily increase in our trial by the increased attention due to a variety of outcome measures. The outcome measures, however, will be given to the participants in all three groups which will make it unlikely for one specific group to experience a larger nonspecific benefit due to the increased attention. We will nevertheless carefully examine whether there will be additional nonspecific benefits in any group and the feasible outcome measures will be selected to work for a main trial. Finally, our study will provide valuable data regarding treatment of AD with acupuncture, but also relative to identifying potential biomarkers associated with its development.

Establishing an adequate sham control is essential in an RCT of acupuncture. We will utilise a nonpenetrating sham acupuncture and nonpenetrating intradermal needles as a control group, which receive sham treatment twice a week for 4 weeks. There has been a long debate about the specific effect of acupuncture: acupuncture works mainly via a placebo effect [70]; or tested sham acupuncture controls so far may not be completely inert [71, 72]. The sham control in our study was designed to minimise a specific physiological effect that may be induced by needle insertion and to avoid specific acupuncture points in the body and the ear. In addition, the number of treatment sessions in the control group was set as twice weekly to minimise other nonspecific benefits such as doctor-patient interaction and the Hawthorne effect; this is feasible due to appropriate blinding techniques.

Attempts have been made to identify biomarkers in order to measure the specific effects of acupuncture and to better understand its mechanism of action [73–75], but no reliable marker has yet to be established due to the lack of sensitivity and/or specificity. Recently, attention has increasingly focused on serum circulating miRNAs as reliable noninvasive or minimally invasive biomarkers because they are abundant, extremely stable in blood, and differentially expressed in various diseases [76]. Hence, we propose exploring novel miRNA-based candidates in association with the antipruritic effect of acupuncture, providing new opportunities to identify valuable biomarkers for the specific effects of acupuncture.

## Supplementary Material

SPIRIT 2013 Checklist: Recommended items to address in a clinical trial protocol and related documents.

## Figures and Tables

**Table 1:** Acupuncture treatment details based on the STRICTA 2010 checklist.

Item	Detail
*(1) Acupuncture rationale*	*(1a) Style of acupuncture* (i) MA and IA using PTN based on traditional meridian theory *(1b) Reasoning for treatment provided, based on historical context, literature sources, and/or consensus methods, with references where appropriate* (i) Partially individualised MA treatments and IA treatments based on the traditional meridian theory, clinical experience, and consensus by the experts in acupuncture and AD *(1c) Extent to which treatment was varied* *MA*: Partially individualised, that is, fixed points plus optional points according to symptoms *IA*: Fixed treatment

*(2) Details of needling*	*(2a) Number of needle insertions per subject per session (mean and range where relevant)* *MA*: From 6 to 19 body acupoints per participant per session *IA*: 2 body acupoints and 1 auricular acupoint per participant per session *(2b) Names (or location if no standard name) of points used (uni/bilateral)* *MA*: (i) Fixed points: PC6, LI11, ST36 bilaterally (ii) Optional points: ST43, GB41, LI2, TE3, TE6, SI2, SI3, BL66, LR3, SP3 contralaterally (GB41, TE3, SI3 can be applied bilaterally according to the signs or symptoms of the patient) *IA*: LI11 bilaterally and auricular shenmen contralaterally *(2c) Depth of insertion, based on a specified unit of measurement or on a particular tissue level* *MA*: From 5 to 30 mm, perpendicular to skin surface *IA*: 1.5 mm, perpendicular to skin surface *(2d) Response sought (e.g., de qi or muscle twitch response)* *MA*: “de qi” sensation *IA*: None *(2e) Needle stimulation (e.g., manual, electrical)* *MA*: Manual stimulation, needle rotation with thumb and index fingers for the first 10–15 seconds *IA*: Participants will be educated to press PTNs for 3 seconds when they feel severe itch *(2f) Needle retention time* *MA*: 15 minutes *IA*: 1-2 days or until PTN falls off *(2g) Needle type (diameter, length, and manufacturer or material)* *MA*: A sterilised stainless steel needle (0.25 × 40 mm, Dongbang Acupuncture Inc., Bundang, Seongnam, Korea) *IA*: A hypoallergenic PTN (1.5 mm, 10 × 10 mm adhesive tape, Haeng Lim Seo Won Medical Co., Korea)

*(3) Treatment regimen*	*(3a) Number of treatment sessions* (i) 8 or 12 sessions according to the allocated group *(3b) Frequency and duration of treatment sessions* (i) Twice or 3 times a week for 4 weeks, 20 minutes for each session

*(4) Other components of treatment*	*(4a) Details of other interventions administered to the acupuncture group (e.g., moxibustion, cupping, herbs, exercises, lifestyle advice)* (i) Lifestyle advices will be given to all participants (ii) Corticosteroids will be allowed as a rescue medicine of severe pruritus through the consensus of the KMD in charge of this study and the pharmacist working for Kyung Hee University Korean Medicine Hospital *(4b) Setting and context of treatment, including instructions to practitioner and information and explanations to patients* (i) A university hospital (ii) Participants will be informed about acupuncture treatment in the study as follows: “In this study, you will be randomly allocated to verum acupuncture treatment or sham acupuncture treatment and acupoints for AD will be selected based on traditional Korean medicine textbook and AD-related reports. Also, additional acupoints can be used according to individual conditions.”

*(5) Practitioner background*	*(5) Description of participating acupuncturists (qualification or professional affiliation, years in acupuncture practice, other relevant experience)* (i) A KMD who has a license and at least two years of clinical experience in dermatologic disorders. He attended 10 hours of training and simulation workshop to ensure that he is able to provide identical acupuncture treatment in accordance with a predefined protocol

*(6) Control or comparator interventions*	*(6a) Rationale for the control or comparator in the context of the research question, with sources that justify this choice* (i) A control group will be treated with sham acupuncture, that is, Park sham acupuncture needles and nonpenetrating sham PTNs *(6b) Precise description of the control or comparator. If sham acupuncture or any other type of acupuncture-like control is used, provide details as for items (1) to (3) above* *MA*: Park sham acupuncture needles and devices will be used in an identical manner as in the verum acupuncture groups. However, fixed acupuncture treatments will be given to 6 control points: a point 1 to 2 cm proximal and 1 cm medial to LI7, a point 1 cm proximal and 1 cm medial to LI11, and a point 1 cm proximal and 1 cm lateral to ST36, each bilaterally *IA*: Nonpenetrating sham PTNs, which were designed and validated for blinding for our study, will be used as a control in an identical manner as in the verum acupuncture groups. The same sized stainless steel rings without needles, that is, nonpenetrating sham, which were designed and validated for blinding for our study, will be attached to three control points: a point 1 cm proximal and 1 cm medial to LI11, bilaterally, and finger point in the ear, contralaterally

AD, atopic dermatitis; IA, intradermal acupuncture; KMD, Korean Medicine Doctor; MA, manual acupuncture; PTN, press tack needle; STRICTA, STandards for Reporting Interventions in Clinical Trials of Acupuncture.

**Table 2:** Study schedule for data collection, treatments, and outcome measures.

Treatment and measures	Baseline	Treatment period				Follow-up period			
		1-week	2-week	3-week	4-week	5-week	6-week	7-week	8-week
Demographics	●								
Physical examination	●								
Medical history	●								
Visual Analogue Scale for itch	○	○	○	○	○		☏		○
SCORAD assessment	○	○	○	○	○				○
EASI assessment	○	○	○	○	○				○
POEM assessment	○	○	○	○	○				○
DLQI assessment	○	○	○	○	○				○
TCS evaluation	○	○	○	○	○		☏		○
AD pattern questionnaire	○								
Cold-Heat questionnaire	●								
PPT evaluation	○		○		○				
CES-D assessment	●				○				○
STAXI assessment	●				○				○
Credibility test	○				○				○
Acupuncture treatment		⊗	⊗	⊗	⊗				
Adverse events		⊗	⊗	⊗	⊗		☏		○
Blood collection	●				○				○

●: conducted on every participant of this study, both AD patients and healthy controls; ○: conducted on AD patients of this study, not on healthy controls; ⊗: conducted on AD patients, but with different frequency on groups. That is, VA1 receives acupuncture treatment or checks adverse events 3 days a week, VA2 2 days a week, and SA 2 days a week; ☏: a telephone interview will be conducted on AD patients of this study, not on healthy controls; AD, atopic dermatitis; CES-D, Center for Epidemiologic Studies-Depression Scale; Cold-Heat, cold-heat pattern identification based on diagnosis in Traditional Korean Medicine; DLQI, Dermatology Life Quality Index; EASI, Eczema Area and Severity Index; POEM, Patient Oriented Eczema Measure; PPT, Pain Pressure Threshold; SCORAD, SCORing Atopic Dermatitis; STAXI, state-trait anger expression inventory; TCS, topical corticosteroid.

## Abbreviations

AD: Atopic dermatitis
AMSRC: Acupuncture and Meridian Science Research Center
ANOVA: Analysis of variance
CES-D: Centre for Epidemiologic Studies-Depression Scale
Cold-Heat: Cold-heat pattern identification based on diagnosis in Traditional Korean Medicine
EASI: Eczema Area and Severity Index
DLQI: Dermatology Life Quality Index
ELISA: Enzyme Linked Immunosorbent Assay
HPA: Hypothalamic-pituitary-adrenal
IA: Intradermal acupuncture
Itch VAS: Visual Analogue Scale for itch
KMD: Korean Medicine Doctor
MA: Manual acupuncture
miRNA: MicroRNA
POEM: Patient Oriented Eczema Measure
PPT: Pain Pressure Threshold
PTN: Press tack needle
SA: Sham acupuncture group
SCORAD: SCORing Atopic Dermatitis
STAXI: State-trait anger expression inventory
STRICTA: STandards for Reporting Interventions in Clinical Trials of Acupuncture
TCS: Topical corticosteroid
VA: Verum acupuncture group
VAS: Visual Analogue Scale
VA1: Verum acupuncture group 1
VA2: Verum acupuncture group 2.
